# The effects of obesity and insulin dysregulation on mare reproduction, pregnancy, and foal health: a review

**DOI:** 10.3389/fvets.2023.1180622

**Published:** 2023-04-20

**Authors:** Isa Hallman, Ninja Karikoski, Maria Kareskoski

**Affiliations:** ^1^Department of Production Animal Medicine, Faculty of Veterinary Medicine, University of Helsinki, Helsinki, Finland; ^2^Department of Equine and Small Animal Medicine, Faculty of Veterinary Medicine, University of Helsinki, Helsinki, Finland

**Keywords:** horse, mare, foal, obesity, insulin dysregulation, equine metabolic syndrome, reproduction

## Abstract

Obesity is a growing welfare concern in modern equine populations and predisposes horses to disturbances in energy metabolism such as insulin dysregulation. However, equine metabolic syndrome has only been recognized in recent decades. Functioning energy metabolism is pivotal to normal body homeostasis and affects essentially all organ systems, including reproduction. Previous literature suggests that obesity has an effect not only on the reproductive processes in mares but also on offspring health, predisposing the offspring to later-onset orthopedic and metabolic problems. This review focuses on the effects of obesity, insulin dysregulation and hyperinsulinemia on the reproductive functions of mares and the implications on foal health before and after birth. The points of interest are the cyclicity and ovarian function, uterine environment, gestation, the postpartum period, and the newborn foal. The aim is to review the current state of knowledge, and identify outstanding questions that could stimulate future research. This topic is important not only from the equine industry and production perspective but is also relevant for the welfare of future populations and individuals.

## Introduction

Obesity is a growing welfare concern in horses, with 24–51% of domestic horses being overconditioned or obese, depending on the breed, season and population (1–4). Obesity is often accompanied by insulin dysregulation (ID) that is strongly linked to equine metabolic syndrome (EMS) and laminitis. Equine metabolic syndrome has been recognized in horses only for recent decades; however, although not extensively studied, the prevalence has been estimated to be as high as 23% in British native breeds (5, 6). Additional to insulin dysregulation, EMS is characterized by general/regional adiposity, predisposition to laminitis, and sometimes dyslipidemia, hypertension, increase in systemic inflammatory markers, and alterations in reproductive function in mares (7).

Nutrition and energy metabolism have been described to affect reproductive and offspring health in many species, including horses (8–14). There are multiple studies describing the negative effects of obesity and ID on fertility, as well as the health of the mare and the growing fetus during pregnancy. Nevertheless, thorough up-to-date reviews are scarce, and the complete effect on reproduction and the offspring are not fully understood (13).

The objective of this review is to describe the current knowledge on the effects of obesity and ID on equine reproduction and health. The review will investigate the different phases of equine reproduction before, during, and after pregnancy. This particular topic is important from a welfare perspective, regarding both the mares and their future offspring. Additionally, as horse breeding is often focused on producing high-functioning animals for sports and recreational use, the long-term effects on the future health and wellbeing of working animals are of special interest to breeders, owners, veterinarians, and increasingly even the general public.

## Overconditioning and obesity in the mare

### The estrous cycle and follicular environment

In the horse, positive energy balance and good body condition have been shown to be mainly beneficial for cyclicity and fertility (15–17). Although obesity and ID are considered welfare issues because of their effects on general health and wellbeing (5, 18, 19), there are contradictory reports on the effect of ID on the reproductive function in horses. While there are similarities between human and equine metabolic disease (5, 18, 20, 21), the effects of obesity on reproduction in horses appears to be quite different and not as debilitating as in people. For example, there is no strong evidence of ovulatory dysfunction or hyperandrogenism due to insulin resistance (IR), hyperinsulinemia, hyperleptinemia or obesity in mares (22, 23). However, in women, it has been shown that anovulatory polycystic ovary syndrome (PCOS) and IR are often associated, and 50% of women suffering from PCOS are overweight or obese (24). In contrast to human PCOS, which typically is accompanied by increased estrogen levels (24), the levels of estradiol in obese mares do not differ from – or are even lower than – mares in moderate or lean condition (25, 26). One possible cause of these differences may be that in women, ovarian steroidogenesis is stimulated by IGF-1, and IR in obese women markedly increases the bioavailability of IGF-1 and subsequently the ovarian steroid production (24). On the other hand, the levels of IGF-1 are not correlated with body condition score (BCS) in mares (25), thus ovarian steroidogenesis or ovulation in mares are not affected by obesity to the same extent as in humans. Plasma LH and FSH concentrations do not appear to differ between IR mares and insulin sensitive mares either (27), and exogenous insulin administration does not affect plasma LH concentrations (28).

In an experiment where insulin sensitive mares were rendered transiently IR with an intravenous lipid infusion, Sessions et al. (29) documented changes in the estrous cycle in the treated mares compared to the control group. They found that experimental IR caused prolonged interovulatory periods and elevated peak progesterone levels during the luteal phase. As the LH concentration remained somewhat unchanged, they concluded that the changes elicited by IR most likely occurred on the level of ovarian function (29). A similar finding was documented by Vick et al. (30), who detected longer duration of estrous cycles and prolonged luteal phases in obese mares. Interestingly, exogenous insulin administration did not affect the cycle length (28), and therefore the effects of ID on the estrous cycle may not be directly due to elevated insulin concentrations. Maternal EMS has been reported to affect the follicular environment and cause altered granulosa cell gene expression, but the clinical relevance of these changes is unclear (31). The question remains whether the described changes in the estrous cycle have any effects on fertility, as fertility outcomes were not studied in the cited reports.

In other studies, mares with high BCS and accompanying hyperinsulinemia have been shown to display normal follicular activity (23). Mares with higher BCS are more likely to continue cycling throughout winter compared to leaner mares, whereas lean mares are more likely to exhibit seasonal anestrus during the winter months (15, 22, 32). If mares with higher BCS are in anestrus during winter, the first ovulation of the season tends to occur earlier compared to lean mares (33, 34). Similarly, it has been reported that younger mares go into anestrus significantly earlier than older mares; this was proposed to be due to higher body condition within the older mare group, and not related to mare age (32).

Leptin is a hormone mainly derived from adipose cells and is, in essence, a biomarker for the degree of adiposity in horses (22, 35, 36). While leptin is essential for normal pubertal and reproductive processes in many species, dysregulation of leptin production and signaling has been proposed to be a participating factor in fertility issues (37). In horses, blood leptin concentrations have been shown to have no effect on fertility, regardless of body condition (38). Even though leptin is traditionally linked to higher body fat content, individual variation exists within different BCS groups (22). Waller et al. (23) studied the variation in leptin concentration within high BCS mares and found no significant difference in ovarian activity during estrous cycle or vernal transition between low leptin and high leptin groups. Sessions-Bresnahan et al. (39) documented increased leptin concentrations in serum and follicular fluid in obese mares, but the clinical relevance of the finding is yet to be determined. Addition of leptin to *in vitro* maturation media caused a significant improvement in oocyte maturation and fertilization after intracytoplasmic sperm injection (ICSI) in horses, but the development of the resulting embryos was significantly reduced compared to the controls (40). Arias-Alvares et al. (41) reported a similar finding with bovine embryos, suggesting that a higher leptin concentration is detrimental to the pre-implantation embryo. In women, increased follicular leptin concentrations have been suggested to cause a lower ovarian response after gonadotropin stimulation compared to leaner individuals, resulting in suboptimal artificial reproduction success, and obese women are known to require higher doses to achieve ovarian hyperstimulation (42). Although ovarian hyperstimulation is not practical or highly successful in horses (43), the correlation documented in women is interesting and raises questions of possible similar tendencies for a reduced responsiveness of ovaries to hormonal treatments in obese animals.

In conclusion, while obesity and IR cause changes in the estrous cycle, according to literature the effect on the resulting fertility may not be entirely deleterious. There is, however, not enough research on the reproductive function in EMS horses to make definite conclusions, and more studies are needed.

### Luteal function

Luteal function and progesterone secretion are vital for normal cyclicity and for the maintenance of early pregnancy in mares (44, 45). In obese women, lipid accumulation in follicles and the ensuing endoplasmic reticulum stress in granulosa cells has been documented to contribute to progesterone deficiency (46). In non-pregnant mares, the concentrations of progesterone and gonadotropins are not correlated with higher leptin concentrations (22). A similar finding was made by Waller et al. (23), who reported that in a group of mares with high BCS, no difference was found in plasma progesterone concentrations between high and low leptin groups. Only 35% of the horses had hyperinsulinemia, and therefore it can be suggested that elevated leptin concentrations are not directly linked to ID/EMS (23). When progesterone concentrations were compared between lean and obese mares, the latter group showed higher levels, which was speculated to be related to regular cyclicity and ovulations or improved luteal function, and no clear adverse effects due to obesity could be determined (25). Another possible contributing factor could be the suggested higher likelihood of obese mares to develop anovulatory follicles, which in turn would lead to persistent luteal structures (30).

It has been reported that experimentally induced, transient IR gives rise to higher peak concentrations of progesterone and a slightly lengthened luteal phase compared to untreated control cycles, but significant differences in mean progesterone concentrations could not be detected (29). However, samples for progesterone were taken only three times per week, which means that the acquired values may not have represented the true progesterone peak. Nevertheless, Sessions et al. (29) concluded that a stimulatory effect of insulin on progesterone production is plausible, as it also supported by findings in other species (47). Exogenous insulin, however, does not influence prostaglandin release or affect luteal size or the length of the diestrus when given to mares daily during days 7 to 17 after ovulation. A tendency to lower progesterone levels was seen but this was thought to be due to a more gradual and earlier onset of luteolysis in the treated mares (28).

Altogether, the published data on luteal function and obesity is rather inconclusive; according to the existing reports, obesity does not seem to markedly alter the function of the corpus luteum.

### Effects on ovulation

Obesity and ID are not considered to be significant predisposing factors for ovulatory failure, which in horses is characterized by anovulation and/or hemorrhage of the dominant follicle, resulting in an infertile cycle and often prolonged interovulatory interval (48–50). Equine metabolic syndrome has been documented to alter the intrafollicular environment. Sessions-Bresnahan and Carnevale (31) speculated that these changes could in turn affect oocyte quality, the maturation process, and ovulation; however, the clinical relevance of their findings remains unclear.

Previous reports describing obesity as a possible predisposing factor for anovulation are conflicting. Vick et al. (30) suggested that obesity is involved in inhibiting or preventing ovulation, leading to formation of anovulatory follicles. In Shetland pony mares fed a high energy diet to induce obesity, the incidence of hemorrhagic anovulatory follicles was higher than in control mares in the first and third years of the study, but lower in the second year. The authors concluded that while ovulatory failure was seen during the study, the obesity-related incidence was inconclusive, and other factors like cycle management with exogenous PGF_2α_, age, breed and individual differences, were more likely to predispose mares to ovulatory failure (34). Elevated LH concentrations have been suggested to predispose mares to the formation of hemorrhagic anovulatory cycles in general (51, 52). However, the concentrations of LH are not affected by IR, insulin concentrations or obesity in mares, so most studies conclude that ID is most likely not the main cause for the emergence of anovulatory follicles (22, 28, 29, 53).

### The non-pregnant endometrium and endometritis

In a recent study, obesity was found to affect the non-pregnant equine endometrium directly, with endometrial progenitor cells showing increases in oxidative stress and early apoptosis in obese mares. However, the clinical relevance of this finding is unclear (54). Apart from this, the effects of metabolic disturbances and obesity on the non-pregnant equine endometrium have not been studied largely. In women, obesity has been strongly linked to endometrial cancer (55), but in horses endometrial neoplasia are rarely reported or diagnosed (56), and to our knowledge, obesity has not been viewed as a predisposing factor. Additionally, reproductive failure in women with PCOS and obesity have been speculated to be the result of a pro-inflammatory environment causing disturbances in insulin signaling pathways within the endometrium (57). As research on the non-pregnant equine endometrium regarding obesity is scarce, it is difficult to make definite conclusions on the uterine health and adiposity.

Endometritis is one of the most common fertility issues in horses, resulting in early embryonic death (EED) and infertility. At least 12.7% of EED cases have been documented to result from endometritis (58, 59), and the condition has been recognized and studied for decades in mares, both as a post breeding-induced inflammatory condition and as a primary bacterial infection of the uterus (59–63). Individual regulation of immunity and inflammation plays an important part in the mare’s susceptibility to the condition: mares more susceptible to persistent breeding-induced endometritis have a different endometrial expression of anti-inflammatory cytokines (IL1RN, IL10, IL6) compared to mares that are not as prone to develop inflammation (64). Obesity and IR are suggested to affect the immune response in horses, but the exact mechanisms are not completely known (65–67). Prolonged systemic levels of IL1β, IL6, IL8, IL10, and TNFα have been demonstrated as a response to endotoxemia in horses with EMS versus non-EMS horses (67). Salinas et al. (68) concluded that obesity alone, without IR or other endocrinological problems, increases the production of reactive oxygen species by neutrophils. Nevertheless, no clear correlation has been reported between body condition and the incidence of endometritis in mares.

## Pregnancy and postpartum period

### Conception and early embryonic death

While obesity in general has not been linked to markedly decreased fertility (15, 69), there are no recent published studies assessing pregnancy rates on EMS horses. In one study on the effects of obesity on embryo transfer results, embryo recovery rate was similar between high BCS and control mares (34), suggesting that elevated body condition does not markedly affect conception rates. However, the report only assessed obesity with a BCS, and the ID status of the ponies was not determined. In a study by Rambags et al. (28), administration of insulin was found to not affect the period of early pregnancy or early equine conceptus. Sessions-Bresnahan et al. (70) documented an obesity-induced increase in endometrial inflammation, as well as changes in the embryo gene expression patterns, endoplasmic reticulum stress, and transcript abundance. They speculated that the changes were most likely due to the altered uterine environment, but also concluded that the magnitude and significance of these changes for the offspring’s future health remain unknown.

Interestingly, D’Fonseca et al. (34) reported that embryos derived from mares fed a high energy (HE) diet were more likely to perish after transfer to either HE or control mares, but when control mare embryos were transferred to HE mares, the post transfer pregnancy rate remained normal. This led to the conclusion that the negative effects on the early pregnancy induced by obesity most likely occur during the follicular stage and early embryo development, rather than due to uterine environment later during development. Therefore, the effect of the altered uterine environment was not perceived as a critically important factor. No differences in embryo diameter or developmental stages were detected between the groups, even though embryo survival rates differed (34).

### Pregnancy-associated IR

A physiological IR develops in mid to late pregnant mares to ensure adequate glucose uptake by the placenta, in order to nourish the growing fetus (71–74). Most pronounced IR has been observed at 7–8 months of gestation, when there is marked fetal bone growth and an increase in mare body weight (74). The alteration is important, as the growing fetus is sensitive to changes in glucose balance: if the maternal glucose levels are compromised, the fetus exhibits an endocrine stress response, with an increased risk of pre-term labor (75). In a study by Beythien et al. (74), insulin release from the pancreas in response to glucose varied according to season in both pregnant mares and geldings, while basal insulin levels remained the same. The authors concluded that these changes enable maximal utilization of glucose at times of scarcity in winter, while also providing efficient modifications of the metabolism to ensure an adequate energy supply to the fetus when the need arises. However, Karikoski et al. (76) concluded that even though the individual ID status and insulin levels in response to oral sugar tolerance test (OST) varied monthly, the variation was not connected to season.

A study from Robles et al. (77) reported that there are also differences in the level of pregnancy- associated IR when comparing primiparous and multiparous mares. The associated IR was lesser in primiparous pregnancies compared to mares with a history of multiple foals, while there was no difference in BCS between the groups (77). A similar effect was detected in women with consecutive pregnancies, however it was postulated that this did not result directly from lower insulin sensitivity due to multiple pregnancies, but rather related to aging and weight gain (78). Primiparous and younger mares were able to feed on higher energy diets without consecutive increase in body mass compared to the older multiparous group (77). It can be speculated that the reasons behind the differences between these two groups may be similar to those in women, suggesting that an increasing number of pregnancies does not necessarily result in a cumulative effect on insulin regulation. This is supported by previous studies, which concluded that basal insulin (79, 80) and the insulin response to both intravenous and oral glucose test in horses increase with age (76, 81).

The direct mechanisms and pathways of IR in pregnancy have not been studied in horses. However, there are multiple reports in women and mice describing the factors contributing to gestational IR (82–84). The factors behind IR during pregnancy and the varying individual magnitude of IR in women include obesity, age, genetic predisposition, gut microbiota, inactivity, PCOS, placental contribution, and hormonal changes (82). Factors inducing the reduction in insulin sensitivity in horses during pregnancy can only be speculated, facilitated by extrapolation from other species. According to equine studies, increasing body mass does not seem to be a marked factor in causing adverse effects to the pregnancy or growing fetus during late gestation. In previous studies, mares with a BCS ≥ 7 have been shown to have similar gestation length, length of parturition, size of foal and placenta and foal viability as mares in moderate condition (16, 85). However, since most of these studies have been conducted without diagnostic testing of insulin status, and BCS only insinuates the possibility of clinical ID or EMS, no certain conclusions can be drawn regarding the effects of ID on late pregnancy.

### Inflammation, obesity, ID and pregnancy

According to previous reviews on EMS, findings on the role of obesity and ID in causing systemic inflammation in the horse have been inconsistent, even though a connection between obesity and inflammation in humans and rodents has been described (7, 86, 87). Vick et al. (65) reported increasing expression of TNFα and IL-1 in mares with increasing BCS, and in a more recent paper Suagee et al. (88) described increasing plasma TNFα and IL-6 during hyperinsulinemia. The acute phase protein SAA has been associated with increasing insulin concentrations as well as BCS (89). However, these findings have not been conclusively supported by all studies (66, 89). More recently, maternal obesity was associated with increased inflammation (with higher levels of TNF and IL1β) and oxidative stress in endometrium (70), as well as changes in the uterine proteome (90) in pregnant mares. The question remains if obesity and metabolic disease affect the uterine environment to the extent of causing harm to the growing embryo.

In women, inflammatory cytokines are known to be involved in the maternal recognition of pregnancy (MRP) and implantation process (91), and obesity has been documented to increase implantation failure (92). In horses, endometrial inflammatory cytokines (IL-6, IL-1β, IL-1α, TNFα) are known to modulate prostaglandin production *in vitro* resulting in either upregulation or downregulation depending on the current hormonal profile and stage of the estrous cycle (93, 94). As prostaglandins are the main component in initiating luteolysis in many species, the role of inflammatory cytokines in these pathways and MRP has been speculated. However, there are no studies elucidating pro-inflammatory cytokine and endometrial interaction during early pregnancy and MRP (44, 95, 96), and with the current knowledge no correlation can be made between metabolic disturbances and MRP in the horse. In previous human studies, it has been proposed that cytokines are actually a contributor to the development of IR in pregnant women – not vice versa (97, 98). In horses, there seems to be an association between cytokine levels and increasing BCS or hyperinsulinemia, but there are no studies on the specific pathways in the development of IR with cytokines as the causative factor (65, 88, 89). Gestation did not increase plasma pro-inflammatory cytokines in mares unless complications, such as placentitis were present (99).

Although late pregnant mares are known to develop physiological IR (73, 74) and in one study, elevated SAA was linked to high plasma insulin concentrations (89), the levels of SAA in healthy pregnant non-ID mares remain low (3.2–8.1 mg/l) before parturition (100). A marked periparturient increase within 36 h postpartum is normally seen in most mares. However, it is presumably not related to metabolic factors, but rather the process of birth itself (100). Robles et al. (101) reported an increase in SAA values in obese mares close to term when compared to mares with normal BCS, and IR was more prominent in the obese group at day 300 of gestation, but no differences were recorded during earlier gestation compared to the control group.

In people, maternal obesity and consecutive IR have been found to cause increases in placental and infant size. However, the size of the placenta did not guarantee high placental efficacy, which was, in fact, negatively correlated with obesity (102). Additionally, an elevation in placental and circulating cytokine levels were detected in obese women but the degree of immune cells within the placental structures was not altered, suggesting that no acute inflammation of the placenta itself was present due to obesity and IR in women (103). In horses, a study by Robles et al. (104) detected that feeding late pregnant mares with cereal concentrates – thus increasing the individual IR – led to an increase in local placental inflammation and alterations in placental structure, such as thickening of the allantoic arterial vascular walls and a reduction in the number of microcotyledonary vessels.

### Laminitis

Painful laminitis is the gravest consequence of ID in horses, and carries serious welfare concerns (86, 105). Mares with ID and/or a history of laminitis may be at higher risk for acute laminitis during pregnancy, as pre-existing conditions are amplified with physiological pregnancy-related IR (106). In a questionnaire by Johnson et al. (106), veterinary practitioners perceived pregnancy as a risk for aggravating a pre-existing tendency for laminitis, especially in obese mares, but not as a direct inciting factor in an otherwise healthy horse. The practitioners also considered laminitis to most likely occur during late gestation (106), which coincides well with the increased IR during that time compared with that of the first trimester (74).

In a study by Pazinato et al. (107), mares with chronic laminitis had shorter gestational length, lower placental weight and lower foal birth weight when compared to healthy mares. Additionally, signs of hypertension and elevated heart rate together with vascular abnormalities in the placenta, such as reduction of the vascular lumen and capillary area in the microcotyledons, thickening of the vascular wall, and fibrosis have been documented (107). These changes are different to vascular findings related to pre-eclampsia in women, which reflects the differences in both the etiopathogenesis of the syndromes and placentation between species (107). The relationship between laminitis and ID has been studied extensively (105), and laminitis during late pregnancy is a recognized problem in the clinical setting (106). Nevertheless, there are no recent publications on laminitis in pregnant mares.

Pain and the concomitant stress response have been shown to lower blood progestagen levels (108, 109) and may therefore predispose the mare to fetal loss especially during early pregnancy (108). Laminitis warrants administration of long-term pain medication in order to accomplish a satisfactory level of comfort for the patient (110). Non-steroidal anti-inflammatories (NSAID) are the most commonly used drug group to alleviate pain, but multimodal analgesia has been described and is becoming more common (110). However, there are no systematic studies on the effects of different pain medication on pregnancy or foal health, and knowledge has mostly been extrapolated from other species (111–115) or accumulated through clinical experience (116). Administration of NSAIDs has been linked to various fetal disturbances and developmental disorders in people, mice, rats and rabbits (111–115), and thus NSAIDs should be used according to careful risk–benefit considerations.

### Obesity, parturition, and postpartum period

In women, maternal obesity has been linked to fetal macrosomia, stillbirth, congenital malformations, and shoulder dystocia (117). Horses, however, do not seem to suffer as gravely from similar consequences of high BCS. The main factor affecting fetal size in horses is the transfer potential of the epitheliochorial placenta. Dystocia due to fetal oversize has traditionally been considered rare, as growth is limited mainly by the surface area of the uterus that is available for placentation (118–121). In most publications, maternal obesity has not been found to markedly affect foal size, foal health, or the mare during birth (15, 85, 122) but Smith et al. (123) described a slight positive correlation between foal birth weight and maternal BCS. According to Rosales et al. (124), overgrowth of the foal was the most common cause for dystocia on a Thoroughbred stud farm. In that study dystocia was defined as the second stage of labor extending over 20 min and birth had to be assisted manually to remove the foal from the birth canal (124). Differences in relation to other reports were suggested to be a result of lengthened gestation (average 350 days), however the BCS of the mares was not assessed (124). In previous reports, longer gestation in Thoroughbreds has not been strongly linked to larger foal sizes at birth (125). The time from parturition to expulsion of the placenta does not seem to differ between healthy mares and mares with chronic laminitis (suggestive of ID/EMS) either (107), but the insulin status or the body condition of these mares were not assessed.

Compared to many other mammalian species, lactational anestrus is not common in mares, as they resume regular cyclicity shortly after parturition if body condition is adequate. The hormonal pathways involved are not completely understood, but lactational anestrus is linked to the suckling behavior and the presence of offspring, which in mares does not suppress the LH surge leading to ovulation (126, 127). Obesity itself does not have a negative effect on postpartal fertility. According to Morley and Murray (128), BCS should be at least 6/9 at the time of foaling to reduce the risk of excessive condition loss postpartum, which would result in worsened fertility. Henneke et al. (15) compared four different groups of pregnant mares. In a group where the mares were allowed to lose body condition prepartum and maintained a low BCS postpartum, the pregnancy results after three cycles were less (50%) than in the other three groups (pregnancy rate 100%) in which mares were either maintained in high BCS, fed to high BCS during postpartum, or allowed to lose BCS postpartum (15). Losing condition already during pregnancy also predisposed the group to EED. As earlier described, overconditioning is more preferable than low condition for fertility, and the degree of overconditioning does not markedly change the situation in horses. When obese (BCS 7 to 8/9) mares were compared to mares in moderate condition (BCS 5 to 6/9) after parturition, the groups had a similar number of days to foal heat ovulation, interovulatory interval, conception rates and maintenance of the following pregnancy (16).

### Lactation

In other species, including humans, it is known that obesity previous to conception or weight gain during gestation can lead to lactation failure, early cessation of lactation, and changes in the udder or milk duct composition and development (129). In horses, the effect of high BCS on lactation is not as marked. Kubiak et al. (130) compared mares with normal BCS to mares fed a high energy diet to the point of obesity during gestation and after parturition. During the lactation period, the normal BCS group tended to show a higher milk yield compared to the obese mares, but the difference between groups was not significant. A recent study by Auclair-Ronzaud et al. (131) reported a higher milk yield in mares with increasing weight, which is contradictory to the previous findings. However, the mares in different groups were of very similar BCS (3.3/5 ± 0.6, and <4/5), therefore increasing weight most likely reflected mare size or height and not obesity. There were no marked differences between milk composition and properties in the mentioned reports, but foals from normal BCS mares tended to be superior in the estimated growth parameters compared to foals from obese mares (130), most likely due to the slightly higher milk yield. Marked fat deposits near the udder of the mare have also been described as a possible prohibitive factor for sufficient suckling behavior (13).

Due to the high energy demand of lactation, pregnant lactating mares generally have lower basal plasma glucose and insulin, as well as a greater insulin sensitivity and glucose tolerance during early pregnancy compared to non-lactating pregnant mares (132). The authors of that study speculated that lactation could enhance the adaptation to metabolic changes, especially during early pregnancy, and attenuate the development of pregnancy related IR. Apart from the study by Kubiak et al. (130), there are not many peer-reviewed studies on obesity, ID and milk yield and composition in mares. Interestingly, mare colostrum has been documented to have a high concentration of insulin at the time of parturition, with a rapid decrease during the first days postpartum. Plasma insulin concentrations of the mares were not measured, and currently both the cause and significance of a prenatal insulin rise close to parturition is not well understood (133).

## The foal

### Uterine environment and the embryo

In studies on various species, it has been proposed that the prerequisites for adult-onset metabolic disease may already be established during uterine development and fetal programming. The maternal metabolic status, body condition, and nutrition during pregnancy can have long-term effects on the metabolic pathways and health of the offspring (9, 10, 134, 135). In horses, maternal IR, inflammation, and enhanced or retarded fetal growth may predispose the foal to various metabolic changes and possible problems with orthopedic health, putting the future career and wellbeing at risk (14, 101, 136). In humans, obese mothers have been shown to be more likely to have infants with higher fat content and predisposition to IR compared to a group of infants from lean mothers (10, 137).

There are few studies on the impact of the uterine environment on the growing fetus in horses. In the equine species, the epitheliochorial placenta mediates the growth capacity provided by the uterine size to the growing embryo (9, 138). For example, Forhead et al. (9) showed that when embryos derived from pony mares were transplanted to Thoroughbred mares, this resulted in an overgrowth of the fetus. The resulting foals had higher basal insulin levels and greater β-cell response to an intravenous glucose stimulus compared to foals from the other breed combinations (pony to pony, Thoroughbred to pony, or Thoroughbred to Thoroughbred). The study shows that overgrowth of the fetus above the norm alters equine β-cell functions and reduces insulin sensitivity. When Thoroughbred embryos were transferred to pony recipients, it resulted in a growth restriction of the fetuses. However, the insulin sensitivity of the growth retarded foals did not differ from the control groups, suggesting that restriction of growth *in utero* does not have as large of an effect on the newborn endocrinological system. A similar effect was seen in a study by Peugnet et al. (136), when pony embryos were transplanted to draft mares, resulting in growth above the original norm of the pony fetuses and increased insulin response to intravenous glucose tolerance test (IVGTT) after birth. Peugnet et al. (136) also transferred Saddlebred embryos to pony, Saddlebred, and draft mares and reported increased insulin sensitivity in the growth restricted transfer group (Saddlebred to pony). Neither of these studies assessed the effect of mare weight or insulin status on the growing embryo, as mare BCS, other obesity measurements or insulin concentrations were not reported. However, Forhead et al. (9) and Peugnet et al. (136) were able to give insight on the underlying importance of the uterine environment and its effect on the offspring.

Sessions-Bresnahan et al. (70) documented oxidative and mitochondrial stress, altered lipid fingerprints and transcript abundance related to lipid homeostasis, inflammation, and endoplasmic retinaculum in embryos derived from obese mares on day 8 and 16, but the clinical significance of the alterations on the offspring remain unclear. Even though changes are evident on the molecular level, a study by D’Fonseca et al. (34) showed that obesity did not affect embryo diameter nor the size of the embryo proper during the early development and up to day 21 of pregnancy.

### Foal size and growth

The size of the neonate foal and factors affecting it have been studied from different angles, but the findings are somewhat conflicting. Robles et al. (77) reported that foals from primiparous mares tend to be smaller than foals from multiparous mares. The degree of IR can be speculated to be one of the reasons for larger foals from multiparous mares; more glucose is left to be transferred to the fetoplacental unit, while the pancreatic response to glucose and insulin sensitivity is reduced. It has also been reported that obese mares generally tend to produce larger foals (123), which coincides with the previously mentioned findings, as both obese and multiparous mares are more likely to have a higher degree of IR (5, 77). However, in studies that assessed the direct relationship between mare overnutrition, obesity and foal birth weight, elevated BCS was not found to result in heavier or taller foals (14, 85, 122, 139), nor did the foal weight or height correlate with the plasma insulin and glucose concentrations of the mare (139).

There are contradictory views on the growth of foals after birth. For example, some studies state that the growth rate after birth is not markedly affected by mare obesity (15, 77, 101). However, Kubiak et al. (130) found that although the weight of foals at birth were similar between obese and normal mare groups, postpartum growth was retarded in foals of obese mares, most likely a result of lesser milk yield. Since neonate size is most likely a multifactorial outcome, assessing the effect of individual aspects is difficult.

### Metabolic alterations in the foal

In humans, high maternal BMI during pregnancy is known to predispose the offspring to cardiovascular and metabolic disturbances and increase the likelihood of obesity, both in the immediate postpartal period and over the long term (140). In horses, there are some studies on the immediate effects of gestational obesity and overnutrition on the foal, but to the authors’ knowledge, long-term studies are lacking. When mares were fed an excess of calories leading to an increase in BCS and reduced insulin sensitivity during late pregnancy, a change in the histoarchitecture of the pancreas was seen in foals immediately after birth, and an increase in the number of islets of Langerhans was reported in the histological analysis. However, no differences in the metabolic hormone levels (insulin, glucagon, and somatostatin) were detected compared to the control group (14). Even without changes in the hormonal panels, the authors speculated that the effect of maternal overnutrition could predispose the foals to metabolic disturbances later in life. However, the surveillance extended only to the period immediately after birth, hence no long-term data is available.

In addition to the amount of feed, its composition has been speculated to elicit changes during pregnancy. George et al. (141) fed late pregnant mares with a high starch (HS) diet and compared the results to pasture-fed mares. The HS foals had a higher baseline glucose concentration compared to the control group, but both groups remained within normal range. Initially there was no difference in insulin sensitivity between the groups, but toward 160 days of age the HS fed group began to show signs of IR. A noteworthy aspect is that neither of the mare groups were overweight (BCS of approximately 6/9) and the BCS did not change markedly during the feeding regimes. Insulin status or other parameters describing the metabolic state of the mares were not assessed (141). Peugnet et al. (142) published a contradictory article, stating that no changes in foal glucose homeostasis were found after the mares were fed a moderate amount of barley during and after pregnancy. The results are not fully comparable, as the feeding regimes and amounts were different, but they do shed light on the interesting aspect of mare to foal changes in glucose homeostasis. The importance of diet has been described in earlier studies, where obesity was not viewed to be as detrimental as the diet itself: mares fed a high energy diet returned to a normal insulin response despite the persistence of obesity, when the energy intake was reduced to normal (143). The alterations in insulin status related to feeding would suggest that in some cases, the feeding regime could be more important than the current body condition of the mare in relation to the foal metabolic health, and even periodical or postprandial hyperinsulinemia could play a role in changing the insulin dynamics in the unborn foal.

Robles et al. (101) documented reduced insulin sensitivity in foals born to obese IR mares for up to 6 months of age, when compared to control foals. However, the difference was lost by the age of 12 months. The authors suggested that the decrease could be related to feeding: foals from IR mares were not as affected by the winter feeding with carbohydrate-rich meals twice per day, which could in turn promote postprandial hyperinsulinemia. No data of the later vulnerability to metabolic changes in these foals was reported (101). Unborn foals are affected by maternal overnutrition, as described earlier, but undernutrition has also been documented to alter the metabolic status of the newborn. Ousey et al. (122) studied five overfed and five moderately conditioned mares that were all subjected to 10% weight loss during mid-pregnancy, and found that the foals from the moderate group had a significantly higher insulin response after glucose administration. This was presumed to indicate alterations in pancreatic β-cell function due to maternal gestational undernutrition, which results in enhanced postprandial hyperinsulinemia. In obese mares with IR, the effect was not detected, and it was speculated that foals from IR mares did not suffer from low glucose and maternal weight loss during gestation. The naturally higher IR related plasma glucose in the obese mare group was suggested to ensure sufficient glucose transport available to the unborn foal and to work as a protective mechanism in times of undernutrition (122).

### Inflammation and orthopedic health

As mentioned previously, inflammatory markers have been suggested to be elevated in horses due to obesity and IR (65, 88, 89). Robles et al. (101) reported low-grade inflammation and elevated levels of SAA until 12 months of age in foals born to obese mares. However, the authors speculated that the elevation of inflammatory parameters in the foals could also be affected by the milk composition or other unknown factors, as the SAA elevation was temporary and the difference between the groups resolved after 12 months (101). In previous studies milk composition (basic nutrients, total solid, fat, protein) has been shown to remain unaltered in mares with gross obesity (130), but milk composition also includes a plethora of other bio-active compounds, which could in theory account for the increases in SAA. Previous studies in women have reported an increase in the inflammatory markers and pro-inflammatory fatty acid profile in breastmilk associated with obesity and gestational weight gain, but the effect on the neonate is not known (144, 145). As far as the authors are aware, no comparable studies have been published in horses.

Orthopedic health is a significant factor affecting the sports performance and career of an individual horse. Maternal nutrition and obesity have been suggested to cause alterations in the fetal cartilage and predispose the foals to orthopedic problems in the future. Additionally, a connection between insulin concentration and equine chondrocyte survival, indicating that insulin may be a factor in osteochondrosis dissecans (OCD) formation in young horses, has been reported (146). Peugnet et al. (142) demonstrated that moderate amounts of barley during pregnancy may predispose the foal to osteochondral lesions at 7 months of age, but the differences failed to reach statistical significance between the study and control groups. Osteochondrosis dissecans is known to be a dynamic state, depending on the joint, and lesions can resolve by the age of 18 months (147). Therefore, the tendency to develop OCD lesions at a younger age can be related to metabolic factors during gestation, but they do not, however, necessarily depict the future orthopedic competence of the foals. Robles et al. (101) studied obese mares and found that gestational obesity could predispose the foals to development of osteochondral lesions at 12 months of age. The difference was significant between the obese and control group at 12 months, but by 18 months, the condition had resolved spontaneously in some of the foals, leading to a non-significant difference between the groups. Altogether 30% of the foals from obese mares had lesions, compared to 10% in the control group; however, the number of the foals in the study was low at this point, which in turn reduced the statistical significance of the finding. The difficulty of studying OCD formation in young horses arises from the fact that OCD is a highly multifactorial disease and therefore more studies with a larger population would need to be conducted to be able to make reliable propositions on the link between gestational obesity and foal OCD.

## Conclusion

Maintaining optimal body condition remains critically important in broodmare management. According to this review, it is clear that obesity has the potential to create changes in both the reproductive function of the mare and the health and athletic outcome of the newborn foal. Since maternal obesity has been documented to cause structural and functional changes in the pancreas of the newborn foal and possibly predispose the neonate to osteochondral lesions later in life, the welfare aspect should not only be focused on the mare. Moderate overconditioning, or obesity without insulin dysregulation seem to be tolerable and even favorable to the mare from a fertility point of view. However, the magnitude and clinical relevance of the changes related to disturbances in insulin regulation described in this review are still uncertain. A noteworthy aspect is that while reproductive success does not seem to be greatly altered, insulin dysregulation may bring other clinical problems, such as laminitis, that are detrimental to the health and wellbeing of the mare.

Although measurable changes have been reported in several parameters related to reproduction, it is challenging to make definite, clinically reliable conclusions on a population level. Acquiring a large enough study population with long enough surveillance time is challenging in equine studies. Additionally, data is often published only on immediate changes and there are no long-term studies on large horse populations to fully elucidate the effect of high BCS or metabolic disturbances on the future generations. Obesity and insulin dysregulation/resistance are often grouped under one term, even though a clear distinction should be made between them, as obesity does not necessarily result in ID, and both can exist without the other. While the effect of obesity and elevated BCS have been studied rather widely regarding reproductive success, essentially only a minority of the studies have evaluated the insulin status of the horses. More studies are warranted to understand the role of ID on the reproductive processes of the mare and the health of future generations.

Obesity and insulin resistance will most likely continue to be a growing concern, as the horse populations in general have experienced a shift from working and athletic sport animals to hobby and leisure horses. One of the most important questions is the possibility of carrying the effects of obesity over generations and predisposing our future equine population to a cycle of disturbed energy metabolism.

## Author contributions

HI and KM were in charge of the initial reviewing of articles and composing the manuscript. KN took part in reviewing and editing the manuscript. All authors contributed to the article and approved the submitted version.

## Conflict of interest

The authors declare that the research was conducted in the absence of any commercial or financial relationships that could be construed as a potential conflict of interest.

## Publisher’s note

All claims expressed in this article are solely those of the authors and do not necessarily represent those of their affiliated organizations, or those of the publisher, the editors and the reviewers. Any product that may be evaluated in this article, or claim that may be made by its manufacturer, is not guaranteed or endorsed by the publisher.
